# On Cultural Differences of Heroes: Evidence From Individualistic and Collectivistic Cultures

**DOI:** 10.1177/01461672221150238

**Published:** 2023-02-02

**Authors:** Yuning Sun, Elaine L. Kinsella, Eric R. Igou

**Affiliations:** 1 University of Limerick, Ireland

**Keywords:** hero, heroism, cultural differences, person perception, prototype analysis

## Abstract

Building on earlier research that examined the characteristics people associate with heroes, our research examined similarities and differences of the hero stereotype across cultures. Specifically, in Study 1 (*N* = 209) and Study 2 (*N* = 298), we investigated lay perceptions of heroes among participants from a collectivistic culture. In Study 3 (*N* = 586), we examined whether group membership could be determined by participants’ centrality ratings of the combined set of hero features. In Study 4 (*N* = 197), we tested whether the hero features that distinguish American and Chinese participants, when used to describe a target person, influence the impression that the target person is a hero. In Study 5 (*N* = 158) and Study 6 (*N* = 591), we investigated cultural differences in perceptions of different types of heroes (e.g., social, martial, civil) and the influence of individualism and collectivism on the perception of those heroes.

Heroes and heroism have attracted the interest of philosophers, artists, and writers for decades (e.g., Campbell, 1949; Carlyle, 1840), but the empirical study of hero–follower relationships has only recently attracted interest in the field of psychology (e.g., Allison & Goethals, 2011; Danilova & Kolpinskaya, 2020; Ulqinaku et al., 2020). Heroes have been described as playing important roles in societies by leading through a crisis and boosting social cohesion (Franco et al., 2011; Kinsella et al., 2015a). Research indicates that heroes provide important psychological functions to individuals by offering psychological and physical protection, enhancing and enriching the lives of others, and modeling morals and values (Kinsella et al., 2015b). These psychosocial functions can be availed of throughout the human lifespan, from childhood through to adulthood (Franco et al., 2011; Kinsella et al., 2015a, 2020; Sullivan & Venter, 2010). During periods of psychological threat or crisis, identification with heroes and heroic attributes becomes more pronounced, suggesting that certain psychological needs are fulfilled by heroes (Coughlan et al., 2019; Kinsella et al., 2015a; Ulqinaku et al., 2020). This has been evident in the increased reporting and celebrating of heroes, such as frontline workers, globally during the Covid-19 pandemic (Cao, 2020; Dimino et al., 2020; Kinsella & Sumner, 2022; Martikainen & Sakki, 2021; Mohammed et al., 2021). So far, researchers have made progress in understanding prototypical features of heroes and also in describing and predicting the psychological functions served by heroes, yet, unfortunately, these have not been examined across cultures.

Culture is a societal-level concept and a group phenomenon that refers to a set of shared ideas about what is valid and valuable in the world (Minkov, 2012; Smith et al., 2013). It is possible that heroes form an important role in representing the values and beliefs within a given culture in a way that is easily translated to all members of the group from an early age through narratives and mythology, and also through modeling of values and behaviors that best represent the group’s success and glory. Indeed, scholars have described heroes as representing society’s most cherished values (Allison & Goethals, 2016). It follows, therefore, that by examining cognitive representations of heroes within a given culture, we will gain new insights into the values and norms that are most valued within that group, as well as how different cultures may use heroes differently for psychological benefit. Despite the rich potential for developing cultural insights from studies of heroes in different groups, there is surprisingly very little knowledge about cross-cultural similarities or differences in perceptions of heroes.

It is a well-known fact that concepts and experiences can vary between cultures (Cross et al., 2014; Lam et al., 2016). Culture is a complex aspect of human behavior, and conducting research across cultures poses many challenges (Milfont & Klein, 2018; Smith, 2010). While some theories of culture have been criticized for being overly simplistic and reductionistic (Greenfield et al., 2003), there is a need to have a basic cultural map of cultures that may be more or less aligned to enable cross-cultural research on everyday concepts to commence. For instance, societies around the world have been roughly mapped onto an individualism–collectivism cultural dimension (Hofstede, 2001). Cultures dominated by individualistic values, such as the United States, emphasize personal goals and self-actualization. In contrast, cultures dominated by collectivistic values, such as China, emphasize collective welfare and common goals (Markus & Kitayama, 1991; Triandis et al., 1988). This distinction between individualistic versus collectivistic cultures (Hofstede, 2001) may provide guidance for conceptualizing potential cultural differences in the perception of heroes.

## Perception of Heroes in Individualistic Cultures

Philosophers and social scientists have offered a multitude of definitions of heroes, but most fail to encompass the complexity and multi-faceted nature of the term. For these reasons, researchers have turned their attention to examining lay conceptions of heroes to understand schematic representations of heroes better. For example, Gash and Conway (1997) investigated the characteristics of heroes among a large sample of children from Ireland and the United States. A list of 24 characteristics of heroes was generated, including being *brave*, *honest*, *strong*, *beautiful*, *loyal*, and *rich*. Sullivan and Venter (2010) conducted a study to understand how the term hero is commonly understood and used by individuals. The findings demonstrated that participants were likely to use a variety of descriptors to define a hero, including *intelligent*, *loving*, *religious*, *caring*, *leader*, *talented*, *hardworking*, *motivated*, *role model*, *kind*, *strong*, and *creative*. Using a similar approach, Allison and Goethals (2011) investigated perceptions of hero characteristics among American college students. They identified eight hero characteristics, including being *strong*, *selfless*, *charismatic*, *resilient*, *inspiring*, *smart*, *reliable*, and *caring*. These lists of characteristics are informative, yet, from these findings, there is no clear sense of which characteristics are most important to our understanding of the hero concept. Thus, to offer more detail and depth, Kinsella et al. (2015a) adopted a prototype approach to identify hero characteristics and to determine the most central (or prototypical) characteristics.

Prototype analyses allow a concept to be defined from a bottom-up lay perspective rather than to be defined from a top-down, specialist, or expert point of view (Gulliford et al., 2020). While top-down approaches can immediately provide the essence of the research based on the researchers’ theorization and critical thinking, they may be too subjective and specific to the research at hand (Luo et al., 2022). Instead, using a bottom-up methodological approach, a prototype analysis provides a scientific approach for understanding multi-faceted concepts that are difficult to define in simple dictionary terms (Morgan et al., 2014). Prototype analyses play an important role in psychological research as it provides greater concept clarity, for example, concerning disillusionment (Maher et al., 2020), gratitude (Lambert et al., 2009), nostalgia (Hepper et al., 2012), and virtue (Gulliford et al., 2020). Hence, a prototype analysis of heroes is not only able to overcome the issues prevalent in top-down approaches, but such analysis is also able to encompass the breadth of conceptions of heroes.

To elucidate lay conceptions of heroes, Kinsella et al. (2015a) examined how individuals think about the features of heroes and how perceptions of heroes differ from other leaders and role models across seven studies. In total, 26 features of heroes were identified via a process of open-ended feature generation and coding. In follow-up studies to assess the relative importance of each of these characteristics, 13 features were identified as central to the hero concept: *brave*, *moral integrity*, *conviction*, *courageous*, *self-sacrifice*, *protecting*, *honest*, *altruistic*, *selfless*, *determined*, *save*, *inspiring*, and *helpful.* The remaining 13 features were considered important to differentiate heroes from other social actors, but more peripheral to the core concept: *proactive*, *strong*, *leader*, *compassionate*, *risk-taker*, *exceptional*, *humble*, *fearless*, *caring*, *powerful*, *intelligent*, *talented*, and *personable.* Furthermore, those researchers found that participants most strongly identified a hero when the target person was described with central (vs. peripheral or neutral) characteristics.

Moreover, there is a consensus that there are three broad forms of heroic types: civil heroes, martial heroes, and social heroes (Franco et al., 2011). Specifically, civil heroes risk themselves to save others from physical harm or death, but there is no training or military code to help them deal with emergencies. An example of a civil hero could be a bystander performing an emergency rescue when someone collapses on the sidewalk (Kinsella et al., 2016). Martial heroes include people who are trained to handle dangerous situations and who are bound to a code of conduct. Examples of martial heroes could be police officers and paramedics (Franco et al., 2011; Zimbardo, 2007). Social heroes typically do not involve an emergency situation but act with courage and kindness to serve or foster their community and its values. An example of a social hero could include a martyr or political leader (Zimbardo, 2007). Previous research in individualistic cultures demonstrated that social heroes are viewed as less heroic than martial heroes or civil heroes (Franco et al., 2011). Researchers have argued that the immediate physical risk related to martial and civil heroes could be an important determining element in the public’s view of heroic types, and thus, it overrides considerations more closely related to social heroes, such as much longer time period and overall risk accepted (Franco et al., 2011; Rate et al., 2007).

## Perception of Heroes in Collectivistic Cultures

While these research findings offer insights into a shared understanding of what a hero means to people, particularly in individualistic cultures, they did not consider the influence of cultural differences on perceptions of heroes. To address this and to examine the cultural limitations of earlier findings, there is a need to consider everyday conceptions of heroes cross-culturally. To our knowledge, there is relatively little empirical work that has attempted to examine lay perceptions of heroes in collectivistic cultures. However, some related studies about heroism in Chinese culture may give us some insights. For example, Chen et al. (2016) found that collectivism was an integral aspect of Chinese superheroes. More specifically, superheroes tend to be perceived as those who are willing to shoulder greater responsibilities to fulfill others’ needs. In a related vein, Zeng and Chan (2020) found that the narratives of heroism in Chinese culture were more likely to emphasize that heroes should sacrifice their own interests to serve collective interests. Zhouxiang and Hong (2019) also argued that top Chinese athletes have usually been regarded as national heroes and they have played an important part in the construction of nationalism and patriotism. It is possible that perceptions of heroes in Chinese culture may be influenced by collectivistic values people hold. However, the perception of heroes in collectivistic cultures has not been systematically examined, and previous findings did not illustrate the influences of individualism and collectivism on the perception of heroes. Thus, our research was designed to fill this void.

## The Present Research

The current project investigated cognitive representations of heroes as a function of cultural differences (individualism vs. collectivism). We conducted six empirical studies. Study 1 investigated the prototypical features of heroes among Chinese participants using open-ended feature generation methods and independent coding. Study 2 examined the centrality of the hero features identified in the previous study through feature rating by independent samples. Study 3 examined the centrality ratings of features of heroes generated by Chinese participants in the current project and features generated by participants from individualistic cultures in Kinsella et al. (2015a). Study 4 examined whether hero features that distinguish between Chinese and American participants (i.e., identified in Study 3) lead to the impression that the target person is considered a hero when used to describe a target person. Study 5 investigated whether there are cultural differences in perceptions of different types of heroes (i.e., martial, civil, social). Finally, Study 6 examined the influence of individualistic and collectivistic values on the perception of those heroes. All studies received ethical approval from the authors’ institution. The study and analyses were not pre-registered. All the data and analyses mentioned in this article have been uploaded on the Open Science Framework: https://osf.io/faz5t/

## Study 1

The aim of Study 1 was to identify prototypical features of heroes in a typical collectivistic (i.e., Chinese) cultural context. Following the format of previous prototype studies (e.g., Kinsella et al., 2015a), we instructed participants to list features of heroes in an open-ended response format.

### Methods

#### Participants

We recruited 209 Chinese participants (140 females; *M*_age_ = 32.85, *SD* = 13.36) via Credamo.^1^ The sample was regionally diverse as participants resided in 107 cities across 24 provinces. This sample size was determined based on the previously published prototype analysis (e.g., Cross et al., 2014; Luo et al., 2022). Each participant was compensated with 2 CNY (around 0.25 Euros) for their participation.

#### Materials and Procedure

Participants were invited to take part in a study about the features of heroes. After consenting to participate, participants were instructed: “In your own view, what are the features that you associate with heroes and heroic actions? There are no right or wrong answers, and please list as many as possible.” The instructions and questions were translated into Chinese and verified by a second translator (a similar procedure was adopted for all studies).

### Results and Discussion

Participants generated a total of 644 exemplars (*M* = 3.08, *SD* = 1.48), composed of either one item from the list or one unit of meaning. All exemplars were translated from Chinese into English following translation and back-translation procedure by proficient individuals (Brislin, 1970). Four independent coders (two Irish and two Chinese), who were blind to the purpose of the study, sorted the original exemplars into superordinate categories by (a) grouping identical exemplars, (b) grouping semantically related exemplars (brave and bravery), and (c) grouping meaning-related exemplars into categories (compassionate and benevolent), a procedure consistent with previous studies (Hepper et al., 2012; Kinsella et al., 2015a). It should be noted that including multiple coders from different cultural backgrounds allows us to rule out the impact of cultural differences during the coding procedure (Regmi et al., 2010). See the Supplemental Material for details.

This resulted in 26 categories subsuming 644 exemplars (see Table 1). In addition, following the procedure in the work of Kinsella et al. (2015a), we asked a fifth (Irish) and a sixth (Chinese) coder independently matched one of the 26 categories (e.g., category: *intelligent*) identified by the four coders to each of the original exemplars (exemplar: *heroes are smart*). There was 88% consistency between the fifth coder’s ratings and the original coding and 83% consistency between the sixth coder’s ratings and the original coding.

**Table 1.** Prototype Features of Heroes, Sample Exemplars, Frequencies (Study 1), and Centrality Ratings (Study 2).

Features	Sample exemplars		Study 1 (*N* = 209)	Study 2 (*N* = 298)	
			Frequencies	*M*	*SD*
Central					
Patriotic	Loves the country	Contributes to the motherland	32	7.19	0.99
Righteous	Being righteous	With a high standard of morality	49	7.16	1.03
Dedicated	Dedication	Devoted	42	7.15	0.98
Brave*	Bravery	Stand up at dangerous moment	64	7.11	1.04
Protect*	Defends weak	Protect people from dangerous situations	28	7.11	1.05
Fearless*	Feeling no fear	Intrepid	27	7.10	0.99
Responsible	Worthy of one’s trust	Has a sense of responsibility	35	7.04	1.06
Respected	Deserves to be respected	Has a good reputation	29	7.04	1.06
Courageous*	Courage	Has the courage to move forward in the face of difficulties	24	7.03	1.04
Helpful*	Helping others	Helps people who are in need	16	7.01	1.11
Moral integrity*	With a high moral integrity	Fair	17	6.99	1.06
Inspiration*	Inspiring	Positive energy	16	6.99	0.98
Determined*	Hardworking	Perseverance	36	6.95	1.10
Peripheral					
Sacrifice*	Sacrifice their own interest for the sake of others	Self-sacrifice	30	6.93	1.11
Save*	Rescue	Saves lives	18	6.93	1.14
Noble	Noble	With the noble spirit	15	6.93	1.13
Selfless*	Puts aside self-interest	Unselfish	32	6.86	1.16
Masculine	Manly	Male figures	14	6.76	1.32
Decisive	Resolute	Act decisively	11	6.73	1.26
Compassionate*	Compassion	Be considerate of others	23	6.62	1.18
Exceptional*	Unique	Excellent	14	6.61	1.32
Intelligent*	Clever	Wise	25	6.50	1.21
Humble*	Modest	Not arrogant	19	6.36	1.39
Altruistic*	Acts for greater good	Altruistic behavior	8	6.15	1.72
Strong*	Strong	Physical strength	11	6.07	1.64
Personable*	Nice	Attractive	9	5.70	1.83

*Note.* Features with * are the features that have also been identified by Kinsella et al. (2015a).

No one feature was mentioned by all participants. There is a great deal of overlap between the features generated by Chinese participants and those previously generated by participants from individualistic cultures (see Kinsella et al., 2015a). Specifically, the 26 features of heroes include 18 features from the original Western sample. This may indicate a cross-cultural consistency of individuals’ understanding of these features. Eight distinctive features were identified: *patriotic*, *righteous*, *responsible*, *respected*, *dedicated*, *noble*, *masculine*, and *decisive*. This may provide evidence that laypeople in collectivistic and individualistic cultures perceive heroes differently, or cultural values may influence their understanding of heroes.

The feature generation task (Study 1) is concerned with implicit measurement, which primarily reflects the ease with which exemplars of heroes spontaneously come to mind (Shi et al., 2021). Next, we examined the importance of these features, which focus on more deliberative, propositional, or reflective aspects of feature identification (Strack & Deutsch, 2004).

## Study 2

This study aimed to assess the centrality of the features that we derived in Study 1 and determine which features were most important in the prototype of heroes among Chinese participants. We instructed an independent sample of Chinese participants to rate how well each of the 26 features related to the concept of heroes.

### Methods

#### Participants

We recruited Chinese participants via Credamo. Initially, we had 300 survey responses. However, two participants failed an attention check question. This left us with a final sample of 298 participants (175 males; *M*_age_ = 29.16, *SD* = 6.40). Each participant was compensated with 2 CNY (around 0.25 Euros) for their participation. The sample size was determined based on previously published prototype analyses (e.g., Cross et al., 2014; Luo et al., 2022).

#### Materials and Procedure

Participants were presented with a list of 26 features in a random order, each accompanied by two exemplars (e.g., the feature *intelligent* was followed by its exemplars *clever* and *wise*). They were asked to rate how closely each of the 26 features related to their personal view of the features of heroes on a scale ranging from 1 (*not at all related*) to 8 (*extremely related*).

### Results and Discussion

As can be seen in Table 1, the ratings for 26 features ranged from 7.19 (Patriotic) to 5.70 (Personable). The median rating was 6.94. Following previous research using prototype analysis, a median split of the centrality ratings was used to divide 26 features into central and peripheral features. The highest 13 central features were: *patriotic*, *righteous*, *dedicated*, *brave*, *protect*, *fearless*, *responsible*, *respected*, *courageous*, *helpful*, *moral integrity*, *inspiration*, and *determined*. The lowest 13 peripheral features were: *sacrifice*, *save*, *noble*, *selfless*, *masculine*, *decisive*, *compassionate*, *exceptional*, *intelligent*, *humble*, *altruistic*, *strong*, and *personable*.

The current findings provide new insight into conceptions of heroes in a collectivistic culture. For instance, while *patriotic* was rated as the most important feature of heroes among Chinese participants, it was not mentioned by participants in previous studies (e.g., Allison & Goethals, 2011; Gash & Conway, 1997; Kinsella et al., 2015a). One reason could be the different cultural values people hold. People from collectivistic cultures are more likely to define themselves as aspects of groups and to give priority to in-group goals (Triandis, 2001). Patriotism, at a group level, fulfills important functions for building group unity and mobilizing individuals to act in ways that will favor their group or country (Sapountzis, 2008). Hence, some exemplars, such as *heroes should love his or her country*, related to the feature of *patriotic*, have been reported frequently by Chinese participants in the current study. In the following studies, we examined cultural differences in conceptions of heroes between Chinese and American participants.

## Study 3

The goal of Study 3 was to examine the centrality ratings of features of heroes across two cultures, including those generated by Chinese participants (generated in Study 1) and features generated by participants in previously published work (see Kinsella et al., 2015a). Specifically, following the study conducted by Joo et al. (2019), we investigated whether group membership (American vs. Chinese) could be reliably determined by the centrality ratings of features generated by both groups of participants and, if so, which features best distinguished the two groups.

### Method

#### Participants

An a prior power analysis revealed a required sample of 536 to have adequate power (.80) to detect a small effect of *f*^2^ = .04 (Cohen, 1988). Taking a conservative approach and allowing for drop-outs and incomplete data, we planned to recruit 600 participants overall. Specifically, we recruited Chinese participants via Credamo. Initially, we had 300 survey responses. However, four participants were excluded as they failed an attention check question (*n* = 2) and failed to complete all questions (*n* = 2). This left us with a final sample of 296 Chinese participants (177 females; *M*_age_ = 30.41, *SD* = 11). Each participant was compensated with 2 CNY (around 0.25 Euros) for their participation. We recruited 300 U.S.-American participants via Amazon Mechanical Turk. However, 10 participants were excluded as they failed an attention check question (*n* = 6) and failed to complete all questions (*n* = 4). This left us with a final sample of 290 U.S.-American participants (146 females; *M*_age_ = 38.07, *SD* = 13.39). Each participant was compensated with US$0.25 for their participation.

#### Materials and Procedure

We combined features of heroes generated in Kinsella et al. (2015a) and those generated by Chinese participants in Study 1 together, leaving 34 features overall. Participants were presented with a list of 34 features in a random order, each accompanied by two exemplars. They were asked to rate how closely each of the 34 features related to their personal view of the features of heroes on a scale ranging from 1 (*not at all related*) to 8 (*extremely related*).

### Results and Discussion

To determine which hero features best distinguish the two groups (i.e., American or Chinese), we conducted a discriminant function analysis (see the Supplemental Material for details). This analysis has been used in various cross-cultural research to predict cultural membership (e.g., Joo et al., 2019; Kashima et al., 1995; Keller et al., 2006). We argued that some hero features would be rated as closely related to a personal view of heroes in one culture but not in the other, thus discriminating between participants from cultures in the United States or China. Those highly endorsed features in one culture represent that those features are closely related to cultural values.

We found that Chinese participants rated *patriotic*, *masculine*, *righteous*, *dedicated*, *responsible*, *respected*, and *noble* as being more related to their personal view of heroes than American participants. In contrast, American participants rated *strong*, *powerful*, *altruistic*, *personable*, *honest*, *leader*, *proactive*, *courageous*, *caring*, and *talented* as being more related to their personal view of heroes than the Chinese participants. Several features did not discriminate well between the groups: *saves*, *humble*, *fearless*, *determined*, *risk-taker*, *moral integrity*, *brave*, *intelligent*, *conviction*, *protects*, *exceptional*, *decisive*, *sacrifice*, *selfless*, *helpful*, *compassionate*, and *inspiration*. In other words, these features were endorsed similarly by the two groups and may represent a common understanding of heroes across both cultures (see Table 2).

**Table 2.** Discriminant Analysis.

Feature	Wilk’s lambda	*F*	Significance
Strong	.72	223.23	*p <*.001
Patriotic	.73	210.56	*p <*.001
Powerful	.74	209.06	*p <*.001
Altruistic	.85	102.60	*p <*.001
Personable	.86	88.63	*p <*.001
Honest	.91	56.32	*p <*.001
Masculine	.93	41.08	*p <*.001
Righteous	.94	35.24	*p <*.001
Leader	.95	25.50	*p <*.001
Proactive	.96	20.82	*p <*.001
Dedicated	.97	16.46	*p <*.001
Responsible	.98	14.28	*p <*.001
Respected	.98	9.82	*p* = .002
Courageous	.98	9.07	*p* = .003
Noble	.99	6.30	*p* = .012
Caring	.99	5.36	*p* = .021
Talented	.99	4.61	*p* = .032
Saves	.99	3.76	*p* = .053
Humble	.99	3.56	*p* = .060
Fearless	.99	3.49	*p* = .062
Determined	1.00	2.59	*p* = .108
Risk-taker	1.00	2.23	*p* = .136
Moral integrity	1.00	1.50	*p* = .222
Brave	1.00	1.28	*p* = .258
Intelligent	1.00	1.19	*p* = .276
Conviction	1.00	.70	*p* = .403
Protects	1.00	.45	*p* = .504
Exceptional	1.00	.30	*p* = .581
Decisive	1.00	.29	*p* = .593
Sacrifice	1.00	.28	*p* = .600
Selfless	1.00	.09	*p* = .761
Helpful	1.00	.04	*p* = .831
Compassionate	1.00	.01	*p* = .909
Inspiration	1.00	.00	*p* = .986

The current findings provide evidence that there are both cultural differences and similarities in lay conceptions of heroes between individualistic and collectivistic cultures. Importantly, of the eight hero features generated by only Chinese participants, Chinese participants rated seven as more related to their personal view of heroes. Of the eight features generated by only participants from individualistic cultures, American participants rated five of them as more related to their personal view of heroes. This may suggest that participants tend to rate the features associated with their cultural values as being more related to their personal view of the features of heroes. In addition, this adds further validity and support to previous studies (e.g., Kinsella et al., 2015a; Sullivan & Venter, 2010) and adds further credibility to the idea of a general prototype of the hero concept across cultures. In Study 4, we further investigated whether participants would more strongly identify a hero when the target was described with the features generated by their respective cultures.

## Study 4

This study aimed to examine further whether hero features that distinguish between Chinese and American participants when used to describe a target person lead to the impression that the target person is considered a hero. Specifically, we expected that participants would evidence a stronger identification of heroes when their cultural features were used to describe the target person.

### Methods

#### Participants and Design

One hundred Chinese participants (48 females; *M*_age_ = 30.23, *SD* = 7.07) were recruited via Credamo. Each Chinese participant was compensated with 2 CNY (around 0.25 Euros) for their participation. We recruited U.S.-American participants via Amazon Mechanical Turk. Initially, we had 100 survey responses. However, three participants were excluded as they failed an attention check question. This left us with a final sample of 97 U.S.-American participants (45 females; *M*_age_ = 39.54, *SD* = 12.64). Each U.S.-American participant was compensated with US$0.25 for their participation. A sensitivity power analysis suggested that the current sample size would detect a small effect size (partial η^2^ = 0.01) using the standard criteria of α = .05 with 80% power. The study followed a 2 (Cultures: American vs. Chinese) × 3 (Conditions: American-related features, Chinese-related features, and Neutral features) mixed design, with cultures as the between-participants variable and conditions as the within-participant variable.

#### Materials and Procedure

Participants were presented with three conditions: American-related features, Chinese-related features, and neutral features. The hero prototypicality of target descriptions was varied across three conditions using the hero features that distinguish between Chinese and American participants and neutral features. Participants were asked to read the descriptions of the target persons carefully.

The target described with hero features that differentiate Chinese conceptions of heroes:

A person showed features of masculinity, dedication, patriotism, righteousness, nobility, and responsibility throughout his or her life. S/he is also respected by many people.

The target described with hero features that differentiate American conceptions of heroes:

A person showed features of strength, power, proactivity, talent, leadership, and courage throughout his or her life. Many people have described her/him as honest, altruistic, personable, and caring.

The target described with neutral features:

A person showed features of stability, assurance, focus and balance throughout his or her life. S/he exerted common sense, rationality, and diligence. S/he also showed purpose and maturity. Many people have described her/him as talkative, reasonable, gentle, and nice.

Participants were asked to think about the persons in the description and rate the extent to which they see the target persons as heroes, from 1 (*strongly disagree*) to 5 (*strongly agree*). Following Kinsella et al. (2015a), four hero-related items were included: the person is a true hero; the person is likely to be seen as a hero; most people would agree that this person is a hero; and in your personal view, based on the description provided, is this person a hero? Cronbach’s αs for the three conditions were satisfactory (.85, .88, and .92, respectively).

### Results and Discussion

We conducted a 2 (Cultures: American vs. Chinese) × 3 (Conditions: American-related features, Chinese-related features, and Neutral features) mixed analysis of variance (ANOVA). Mauchly’s test of sphericity was significant, χ^2^(2) = 14.67, *p* = .001, indicating that the Greenhouse–Geisser estimates with the corrected degrees of freedom would be appropriate.

**Figure 1.**
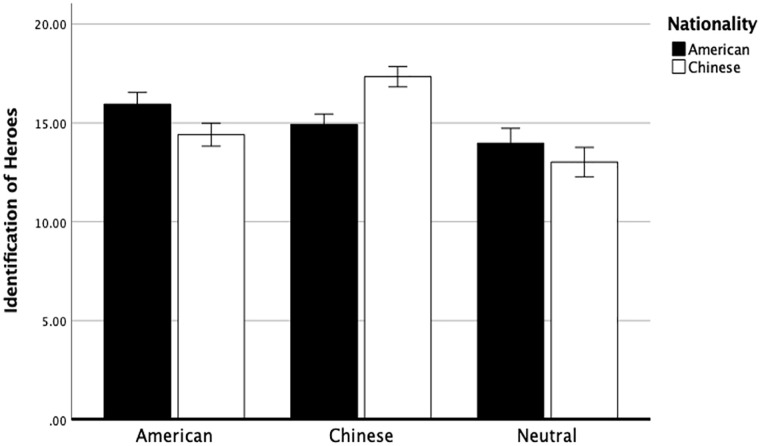
Identification of Heroes. *Note.* The four hero-related items were aggregated as a sum. Error bars represent 95% confidence interval.

As predicted, there was a significant interaction between cultures and conditions on identification of heroes, *F*(1.86, 363.53) = 36.97, *p* < .001, partial η^2^ = .16. Importantly, follow-up tests revealed that American participants (*M* = 15.95, *SD* = 2.82) scored higher on American-related features condition than Chinese participants (*M* = 14.41, *SD* = 3.10), *F*(1, 195) = 13.28, *p* < .001, partial η^2^ = .06. Chinese participants (*M* = 17.34, *SD* = 2.17) scored higher on Chinese-related features condition than American participants (*M* = 14.93, *SD* = 2.17), *F*(1, 195) = 41.91, *p* < .001, partial η^2^ = .17. There were no significant differences between Chinese participants (*M* = 13.02, *SD* = 4.05) and American participants (*M* = 13.98, *SD* = 3.45) on neutral conditions (see Figure 1).

These findings suggest that participants were likely to more strongly identify a hero when their cultural features were used to describe the target person. This also reinforces the idea that there are some cultural differences in perceptions of heroes. To further build on this work, in the next study, we examine cultural differences in the perception of different types of heroes.

## Study 5

In Study 5, we examined cultural differences in three types of heroes: civil heroes, martial heroes, and social heroes. Previous research demonstrated that social heroes are viewed as less heroic than martial heroes or civil heroes (Franco et al., 2011). However, previous studies did not consider the influence of cultural differences on perceptions of different types of heroes. As mentioned previously, the narratives of heroism in Chinese culture tend to emphasize that heroes should sacrifice their own interests to serve one’s country and focus on the construction of nationalism and patriotism (Chen et al., 2016; Zeng & Chan, 2020). The findings from Studies 1–4 demonstrated that Chinese participants were more likely to use *patriotic* to define a hero compared with American participants.

We pose that perceptions of heroes are influenced by cultural values people hold. Specifically, we expect Chinese participants to perceive heroes that involve bringing about social changes to a country or laying the foundations of new values and principles (i.e., social heroes) as more heroic than the other two types of heroes. In contrast, based on the previous findings (e.g., Franco et al., 2011), American participants would perceive social heroes as being less heroic compared with the other two types of heroes.

### Methods

#### Participants and Design

Eighty-one Chinese participants (48 males; *M*_age_ = 28.54, *SD* = 4.62) were recruited via Credamo. Each Chinese participant was compensated with 2 CNY (around 0.25 Euros) for their participation. Seventy-seven U.S.-American participants (45 males; *M*_age_ = 38.21, *SD* = 12.97) were recruited via Amazon Mechanical Turk. Each U.S.-American participant was compensated with US$0.25 for their participation. A sensitivity power analysis suggested that the current sample size would detect a small effect size (partial η^2^ = 0.01) using the standard criteria of α = .05 with 80% power. The study followed a 2 (Cultures: American vs. Chinese) × 3 (Conditions: Civil Heroes, Martial Heroes, and Social Heroes) mixed design, with cultures as the between-participants variable and conditions as the within-participants variable.

#### Materials and Procedure

Participants were presented with three vignettes developed by the research team for the purpose of this study: civil heroes, martial heroes, and social heroes. The hero prototypicality of target descriptions was the same across three conditions using the hero features identified by both Chinese and American participants. Target persons were left unidentified to eliminate the risk of contamination of prior knowledge about targets in this task. Participants were asked to read the descriptions of the target persons carefully. The three vignettes are presented below.

##### Civil Heroes

*Person A showed the features of bravery, selflessness, and fearlessness throughout her or his life. S/he also showed a willingness to protect and save others. Many people have described Person A as self-sacrificing. After a 58-year-old person fell onto subway tracks on Saturday night, Person A jumped onto railway tracks to lift the person to safety. “People were screaming to get help,” Person A said after the incident. “But nobody jumped down. So I jumped down.”*

##### Martial Heroes

*Person B showed the features of bravery, selflessness, and fearlessness throughout her or his life. Person B also showed a willingness to protect and save others. Many people have described Person B as self-sacrificing. Person B worked at the front line for countless families. “I’m one of those who could have retired, but I chose not to. We all need to do what we can, and what we can do best, to deal with it.”*

##### Social Heroes

*Person C showed the features of bravery, selflessness, and fearlessness throughout her or his life. Person C also showed a willingness to protect and save others. Many people have described Person C as self-sacrificing. Person C led a successful campaign for the country’s independence and, in turn, inspired movements for civil rights and freedom across the world.*

Participants were asked to think about the persons in the description and rate the extent to which they see target persons as heroes from 1 (*strongly disagree*) to 5 (*strongly agree*). In line with the work of Kinsella et al. (2015a), four hero-related items were included: the person is a true hero; the person is likely to be seen as a hero; most people would agree that this person is a hero; and in your personal view, based on the description provided, is this person a hero? Cronbach’s αs for the three conditions were satisfactory (.76, .86, and .85, respectively).

### Results and Discussion

**Figure 2.**
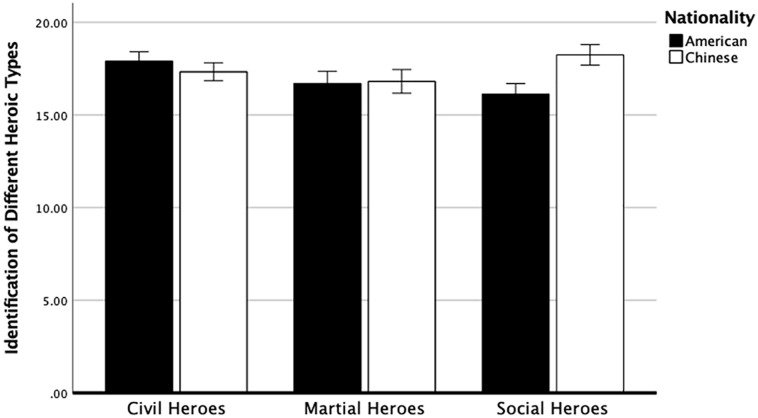
Identification of Different Heroic Types (Study 5). *Note.* The four hero-related items were aggregated as a sum. Error bars represent 95% confidence interval.

We conducted a 2 (Cultures: American vs. Chinese) × 3 (Conditions: Civil Heroes, Martial Heroes, and Social Heroes) mixed ANOVA. Mauchly’s test of sphericity indicated that the assumption of sphericity had been met, χ^2^(2) = 5.25, *p* = .072. There was a significant interaction between cultures and conditions on the identification of different heroic types, *F* (2, 312) = 17.69, *p* < .001, partial η^2^ = .10 (see Figure 2). Follow-up tests revealed that scores were higher in the social heroes’ condition among Chinese participants (*M* = 18.25, *SD* = 1.72) than American participants (*M* = 16.13, *SD* = 3.13), *F*(1, 156) = 28.05, *p <* .001, partial η^2^ = .15. However, there were no significant differences between Chinese and American participants on either civil or martial heroes’ condition. Furthermore, among American participants, scores were higher in the civil heroes’ condition (*M* = 17.91, *SD* = 2.37) compared with the martial heroes’ condition (*M* = 16.70, *SD* = 3.10; *p* = .002) and the social heroes’ condition (*M* = 16.13, *SD* = 3.13; *p* < .001). Among Chinese participants, scores were higher in the social heroes’ condition (*M* = 18.25, *SD* = 1.72) compared with the civil heroes’ condition (*M* = 17.33, *SD* = 2.05; *p* = .008) and the martial heroes’ condition (*M* = 16.82, *SD* = 2.73; *p* < .001).

These findings strongly confirm cultural differences in perceptions of *different types* of heroes (i.e., civil, martial, social) and provide further evidence that cultural values may influence the understanding of heroes. In Study 6, we examined whether individualism (vs. collectivism) predicted the perception of *different types* of heroes.

## Study 6

The aim of Study 6 was to investigate the effects of individualism and collectivism on perceptions of heroes. Importantly, Study 6 extends Study 5 in two ways. First, we focused only on the *behaviors* of different types of heroes due to the fact that in Study 5 it was not clear if participants’ responses reflected the features of heroes (e.g., *bravery*, *selflessness*, *and fearlessness*) or heroic behaviors (e.g., *Person A jumped onto railway tracks to lift the person to safety*). Second, we examined to role of individualistic and collectivistic values in the identification of different types of heroes.

It should be noted that an individual can hold both individualistic and collectivistic values (Triandis, 2001), and it is important to identify which domain of values is most likely to be related to the phenomenon being studied to make clear and accurate conclusions (Brewer & Chen, 2007). We argue that the effects of individualism (vs. collectivism) on the identification of heroes may be different for American and Chinese participants. For example, people who endorse collectivistic values think of people as highly interconnected to one another and describe others as embedded in a special social context (Markus & Kitayama, 1991; Nisbett et al., 2001). Hence, collectivistic values may be more influential in predicting the identification of heroes among Chinese participants and Chinese participants may tend to prioritize collective interests (e.g., serving the country) that heroes could serve. In contrast, participants from individualistic cultures tend to emphasize personal causes of social behaviors, such as traits (Nisbett et al., 2001). For example, Kinsella et al. (2015a) found that *brave* was the most prototypical feature of heroes rated by participants from individualistic cultures, and we found that American participants tend to perceive civil heroes as being more heroic compared with other types of heroes, as civil heroes hold no situation training and strongly emphasize personal characteristics, such as bravery. Therefore, individualistic values may be more influential in predicting the identification of heroes among American participants. We expected that the effects of individualism (vs. collectivism) on the identification of heroes (particularly on the identification of social heroes) would differ between American and Chinese participants, which could also explain the cultural differences in the identification of social heroes.

### Methods

#### Participants and Design

Two hundred and ninety-seven Chinese participants (146 males; *M*_age_ = 35.44, *SD* = 8.48) were recruited via Credamo. Each Chinese participant was compensated with 2 CNY (around 0.25 Euros) for their participation. Two hundred and ninety-four U.S.-American participants (170 males; *M*_age_ = 36.49, *SD* = 11.49) were recruited via Amazon Mechanical Turk. Each U.S.-American participant was compensated with US$0.25 for their participation. A sensitivity power analysis suggested that the current sample size would detect a small effect size (partial η^2^ = 0.01) using the standard criteria of α = .05 with 80% power. The study followed a 2 (Cultures: American vs. Chinese) × 3 (Conditions: Civil Heroes, Martial Heroes, and Social Heroes) mixed design, with culture as the between-participants variable and conditions as the within-participants variable.

#### Materials and Procedure

Participants were presented with three vignettes.

##### Civil Heroes

*After a 58-year-old person fell onto subway tracks on Saturday night, Person A jumped onto railway tracks to lift the person to safety. “People were screaming to get help,” Person A said after the incident. “But nobody jumped down. So I jumped down.”*

##### Martial Heroes

*Person B worked at the front line for countless families. “I’m one of those who could have retired, but I chose not to. We all need to do what we can, and what we can do best, to deal with it.”*

##### Social Heroes

*Person C led a successful campaign for the country’s independence, and in turn, inspired movements for civil rights and freedom across the world.*

Participants were asked to think about each person in the descriptions and rate the extent to which they viewed each target person as a hero from 1 (*strongly disagree*) to 5 (*strongly agree*). As in Study 5, four hero-related items were included. Cronbach’s αs for the three conditions were satisfactory (.76, .88, and .89, respectively). In addition, participants completed Oyserman’s (1993) six-item individualism (e.g., “I determine my own destiny,” α = .95) and six-item collectivism (e.g., “In general, I accept the decisions made by my group,” α = .94) scales (1 = *strongly disagree*, 7 = *strongly agree*).

### Results and Discussion

#### Cultural Differences in Perception of Heroes

**Figure 3.**
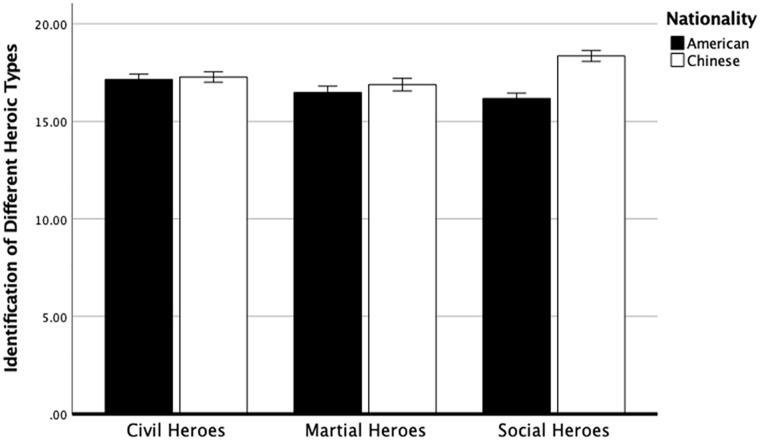
Identification of Different Heroic Types (Study 6). *Note.* The four hero items were aggregated as a sum. Error bars represent 95% confidence interval.

Overall, we replicated the findings from Study 5 (see Figure 3). There was a significant interaction between cultures and conditions on the identification of different heroic types, *F* (2, 1,178) = 41.59, *p* < .001, partial η^2^ = .06. Scores were higher in the social heroes’ condition among Chinese participants (*M* = 18.35, *SD* = 1.78) compared with American participants (*M* = 16.17, *SD* = 2.97), *F*(1, 589) = 117.16, *p* < .001. partial η^2^ = .16. There were no significant differences between Chinese and American participants on either civil or martial heroes’ conditions. Furthermore, among American participants, scores were higher in the civil heroes’ condition (*M* = 17.15, *SD* = 2.19) than in the martial heroes’ condition (*M* = 16.48, *SD* = 3.08; *p* < .001) and the social heroes’ condition (*M* = 16.17, *SD* = 2.97; *p* < .001). Among Chinese participants, scores were higher in the social heroes’ condition (*M* = 18.35, *SD* = 1.78) than in the civil heroes’ condition (*M* = 17.28, *SD* = 2.56; *p* < .001) and martial heroes’ condition (*M* = 16.89, *SD* = 2.64; *p* < .001).

#### Effects of Individualism and Collectivism on Perception of Heroes

We found Chinese participants (*M* = 33.50, *SD* = 5.32) reported a greater level of collectivism than American participants (*M* = 28.70, *SD* = 9.81), *t*(450) = −7.38, *p* < .001, *d* = −.61, 95% CI [−.77, −.44]. American participants (*M* = 33.05, *SD* = 5.94) reported a greater level of individualism than Chinese participants (*M* = 26.05, *SD* = 7.66), *t*(557) = 12.42, *p* < .001, *d* = 1.02, 95% CI [0.85, 1.19]. This is consistent with findings in cultural differences in individualism and collectivism reported by previous studies (e.g., Kobayashi et al., 2021; Liu et al., 2021; Santos et al., 2017). See Tables 3 and 4 for correlations of key variables.

**Table 3.** Correlations of Key Variables Among Chinese Participants.

Variable	1	2	3	4	5
Civil heroes	—				
Martial heroes	.260***	—			
Social heroes	.180**	.256***	—		
Individualism	.442***	.218***	−.082	—	
Collectivism	.102	.054	.172**	.023	—

*Note*. *p < .05. **p < .01. ***p < .001 (two-tailed).

**Table 4.** Correlations of Key Variables Among American Participants.

Variable	1	2	3	4	5
Civil heroes	—				
Martial heroes	.456***	—			
Social heroes	.368**	.448***	—		
Individualism	.413***	.467***	.341***	—	
Collectivism	−.199***	−.171**	−.095	−.145*	—

*Note*. *p < .05. **p < .01. ***p < .001 (two-tailed).

We regressed the identification of three types of heroes (i.e., civil heroes, martial heroes, and social heroes) on individualistic and collectivistic values. We found individualism predicted identification of civil heroes, β = .37, *F*(1, 588) = 90.82, *p* < .001, and martial heroes, β = .26, *F*(1, 588) = 39.64, *p* < .001, after controlling for collectivism; individualism did not predict identification of social heroes, β = −.05, *F*(1, 588) = 1.50, *p* = .22, after controlling for collectivism. In addition, collectivism predicted identification of social heroes, β = .08, *F*(1, 588) = 4.04, *p* = .04, after controlling for individualism; collectivism did not predict identification of civil heroes, β = .01, *F*(1, 588) = .03, *p* = .86, and martial heroes, β = −.02, *F*(1, 588) = .30, *p* = .58, after controlling for individualism.

#### Additional Analyses

We conducted a series of moderation analyses (PROCESS Model 1; Hayes, 2017) to examine the interaction between cultural group and individualism/collectivism (see the Supplemental Material for full details). Importantly, we found a significant interaction between cultural group and individualism on the identification of social heroes (see Table 5), *B* = .187, 95% CI [.095, .272], *t* = 6.42, *p* < .001. Further tests revealed that higher levels of individualistic values significantly predict higher levels of identification of social heroes among American participants (*B* = .17, *t* = 7.42, *p* < .001, 95% CI [.13, .22]) but not among Chinese participants (*B* = −.02, *t* = −1.07, *p* = .286, 95% CI [−.05, .02]). We also found a significant interaction between cultural group and collectivism on the identification of social heroes (see Table 6), *B* = −.072, 95% CI [−.128, −.012], *t* = −2.49, *p* = .014. Further tests revealed higher levels of collectivistic values predicted higher levels of identification of social heroes, *B* = .06, *t* = 2.17, *p* =.031, 95% CI [.005, .11], among Chinese participants and higher levels of collectivism predicted lower levels of identification of social heroes, *B* = −.03, *t* = −1.99, *p* = .047, 95% CI [−.06, −.0004], among American participants.

**Table 5.** Collectivism Predicting Identification of Social Heroes.

Predictors	*B*	*SE*	*t*	*p*	95% CI
Constant	18.21	.16	117.52	< .001	[17.91,18.52]
CO	.06	.03	2.17	.03	[.005, .11]
CG	−2.11	.21	−9.90	< .001	[−2.53, −1.69]
CG × CO	−.09	.03	−2.85	.005	[−.15, −.03]

*Note. R*^2^ = .18, *F* (3, 587) = 42.38, *p* < .001; *R*^2^ *change* = .01, *F* (1, 587) = 8.15, *p* = .004. CG = cultural group (0 = Chinese, 1 = American); CO = collectivism; CI = confidence interval.

**Table 6.** Individualism Predicting Identification of Social Heroes.

Predictors	*B*	*SE*	*T*	*p*	95% CI
Constant	18.29	.15	122.43	< .001	[17.99, 18.58]
IN	−.02	.02	−1.07	.29	[−.05, .02]
CG	−2.72	.22	−12.45	< .001	[−3.14, −2.29]
CG × IN	.19	.03	6.53	< .001	[.13, .25]

*Note. R*^2^ *=* .24, *F* (3, 587) *=* 61.37, *p* < .001; *R*^2^ *Change =* .06, *F* (1, 587) *=* 42.58, *p <* .001. CG = cultural group (0 = Chinese, 1 = American); IN = individualism; CI = confidence interval.

We found that the perception of social heroes (e.g., martyrs, political figures, and religious leaders who lead a nation or inspire a movement for civil rights and freedom) is associated with collectivistic value orientations among Chinese participants. For example, people from collectivistic cultures (e.g., China) are encouraged to link their lives with the destiny of the nation and to dedicate themselves to the country (Bai, 2022; Naftali, 2021), and people may be more sensitive to heroic actions that prioritize the collective over individual interests (e.g., collectivistic features of social heroes). In contrast, people from individualistic cultures (e.g., the United States) may be more sensitive to the personal causes of social heroes, such as their individualistic characteristics. Compared with other types of heroes, social heroes strongly emphasize collectivistic features, and they incorporate features (e.g., loyalty to the country and willingness to sacrifice their own interests for the country) that are strongly related to values that Chinese people tend to adopt. Hence, Chinese participants are likely to perceive social heroes as being more heroic than the other two types of heroes, and they are expected to score higher in the social heroes’ condition than American participants.

Heroes are often recognized for their agentic characteristics, such as their bravery and determination, as well as their communal characteristics, such as a sense of community and the willingness to sacrifice for the greater good (see Kinsella, Ritchie, & Igou, 2017). The perception of heroes may be shaped by various characteristics that people tend to focus on, which are often linked to their cultural values. Therefore, it is important to consider both individualistic and collectivistic values when investigating cultural differences in the perception of heroes, rather than considering them in isolation. For example, our findings also demonstrated higher levels of individualistic values predict the identification of civil and martial heroes among both American and Chinese participants, as both civil and martial heroes strongly emphasize on individualistic features and prominent individualistic heroic actions (e.g., a person jumped onto railway tracks to lift the person to safety).

## General Discussion

The aims of the present research were to examine whether perceptions of heroes differ between individualistic and collectivistic cultures and to examine how cultural values predict the identification of hero features and different types of heroes. In Studies 1 and 2, we systematically identified 26 features of heroes among Chinese participants and examined the centrality (i.e., importance or prototypicality) of each of these features. In four subsequent studies, we demonstrated which features best distinguish Chinese and American participants (Studies 3 and 4), cultural differences in perceptions of different types of heroes (Study 5), and whether individualism and collectivism predicted the perceptions of heroes (Study 6).

These studies represent an important step toward understanding conceptions of heroes in a collectivistic culture and how cultural values influence the identification of heroes. Human societies differ in a variety of psychological and behavioral tendencies (Varnum & Grossmann, 2017), and therefore, it is interesting to explore how we conceptualize heroes in different societies. Importantly, examining lay conceptions of heroes in a non-Western culture can be helpful for contributing to understanding how heroes are used in everyday life in diverse cultures and promoting hero-related education initiatives. For example, the Heroic Imagination Project (Zimbardo, 2007) was set up to promote everyday heroism and encourage people to be kinder, more prosocial, and more effective in dealing with unclear or emergency situations. Thus, on one hand, it is possible to translate the central and peripheral features of heroes identified by the current study and the previous study into accessible terms to guide heroic development and provide a roadmap for personal development (Kinsella et al., 2016). On the other hand, however, when delivering hero-related education programs in collectivistic cultures, consideration must be given to the conceptions of heroes with an awareness of cultural differences. For instance, as *patriotic* is the most prototypical feature of heroes rated by Chinese participants, Chinese people may think real heroism is not available to everyone but is only available for those people who have significant contributions to the country.

Second, the current findings contribute to a deeper understanding of how culture shapes perceptions and that using the psychology of heroism helps to understand human behavior within and across cultures. For example, heroes are often recognized for helping individuals to find meaning and purpose in daily life (Kinsella et al., 2015b). Researchers found that individuals first appreciate the exceptional efforts of heroes, which, in turn, translates into a personal desire to search for meaning in life (Coughlan et al., 2019; Kinsella, Igou, & Ritchie, 2017). However, are there cultural differences in this psychological process? Is it possible that culture influences the perceptions of heroes, which in turn influences individuals’ motivation to search for more socially-oriented (vs. individual-oriented) meaning in life or happiness (Datu et al., 2016; Hitokoto & Uchida, 2015; Lu & Gilmour, 2006?)? Future research should examine the potential influence of culture in this relationship. Furthermore, this could also lead to future research about perceptions of whistleblowers across cultures. Whistleblowers are those individuals who expose information or activities that are deemed illegal or unethical in an organization or government, and their actions are often viewed as heroic (Franco et al., 2011). However, people from collectivistic cultures (such as Japan and China) view whistleblowing less favorably than individuals from individualistic cultures (Brody et al., 1999). Researchers argued that this cultural difference is due to a culture’s degree of collectivism. For example, participants who endorse collectivistic values would perceive group harmony as more important and, thus, would express more negative feelings toward whistleblowing (Dungan et al., 2015). Future studies may consider if the perceptions of whistleblowers could also be influenced by patriotism, as their actions may diminish the reputation of the country, and thus whistleblowing would be viewed more negatively.

Third, our work makes important contributions to the development of methodology in cross-cultural psychology by using a prototype analysis to examine cultural differences in lay understandings of a concept. Wang (2016) argued that it is important to take advantage of the unique methodological tools in cross-cultural psychology to examine not only group differences but also highlight the active role of individuals in shaping cultural differences. Hence, from the methodological point of view, using the data-driven, bottom-up approach to collect lay conceptualization of heroes gives voice to the participants as active producers of definitions of heroes rather than based on researchers’ assumptions and expectations. Furthermore, this approach allows for the inclusion of additional features that represent lay people’s definitions of heroes in a non-Western culture.

However, our project contains some limitations. First, we have only identified features of heroes among Chinese participants. China is the largest Eastern country known for collectivism (Oyserman et al., 2002). Cross-cultural studies typically examine individuals from China as representatives of Eastern and collectivistic cultures (Feinberg et al., 2019; Lin et al., 2022). Thus, our research offers an important first step toward understanding conceptions of heroes in Eastern culture. However, it should be noted that there may be differences within China (e.g., urban vs. rural areas; Talhelm et al., 2015). In addition, although China is a typical collectivistic society, replication of our findings in other Eastern or collectivistic countries would help capture a broader picture of the perception of heroes. Second, following previous studies (e.g., Joo et al., 2019; Shi et al., 2021), we used the first two steps in the prototype approach to generate features of heroes and assess the centrality of these features. It may be possible for future research to include additional measures of prototypicality (e.g., reaction times).

Our research examined differences in the perception of heroes between American and Chinese participants. The findings demonstrate essential cultural differences in the perception of features of heroes and how individualistic and collectivistic values influence perception of different types of heroes. These studies offer an important first step toward understanding cultural differences in perceptions of heroes in individualistic and collectivistic cultures and provide a crucial foundation for further development and application of research on heroes and heroism.

## Supplemental Material

sj-docx-1-psp-10.1177_01461672221150238 – Supplemental material for On Cultural Differences of Heroes: Evidence From Individualistic and Collectivistic Cultures Supplemental material, sj-docx-1-psp-10.1177_01461672221150238 for On Cultural Differences of Heroes: Evidence From Individualistic and Collectivistic Cultures by Yuning Sun, Elaine L. Kinsella and Eric R. Igou in Personality and Social Psychology Bulletin

## Notes

1. A Chinese data-collection platform is similar to Amazon Mechanical Turk (https://www.credamo.com).
