# Supplementary material for: On Cultural Differences of Heroes: Evidence From Individualistic and Collectivistic Cultures
Source: Pers Soc Psychol Bull. 2023 Feb 2;50(6):841–56. doi: 10.1177/01461672221150238 (PMC11080389; doi:10.1177/01461672221150238)
Supplement: sj-docx-1-psp-10.1177_01461672221150238 – Supplemental material for On Cultural Differences of Heroes: Evidence From Individualistic and Collectivistic Cultures [file sj-docx-1-psp-10.1177_01461672221150238.docx]

**Supplementary Materials**

**On Cultural Differences of Heroes:** **Evidence from Individualistic and Collectivistic Cultures**

**Study 1**

The first Irish coder identified 19 categories, and the second Irish coder identified 27 categories. There was 60% agreement between the two coders. In addition, the first Chinese coder identified 26 categories, and the second Chinese coder identified 19 categories. There was 66% agreement between the two coders. Following the procedure reported in previous prototype analyses (e.g., Joo et al., 2019; Neto & Mulle, 2014), the researcher and four coders engaged in further discussions to resolve any discrepancies to ensure the validity of the coding. Specifically, all coders agreed to combine *aspiring* into *determined*, *rational* into *intelligent*, as they were conceptually similar. All coders agreed splitting *brave*and *courage* into distinct categories, as the meaning of *brave*, and *courage*is different in both English and Chinese language (Cheng & Huang, 2017; Kinsella et al., 2017). In addition, a few exemplars were Chinese idioms. Although they have superficial lexical dissimilarity, they were semantically equivalent, providing alternative ways to convey the same idea (Shi et al., 2020). For example, *able to hold up heavens and support the earth* has the meaning of being masculine in Chinese (Jiao et al., 2013). Hence, rather than categorizing it as *superpower*, four coders all agreed to combine it into *masculine*.

**Study 2**

**Reliability of Ratings**

A two-way mixed-effects intra-class (ICC) was conducted to test the reliability of these ratings. Twenty-six features were treated as cases, and the 298 participants were treated as items. The analysis revealed that participants’ ratings of the features were very reliable (ICC= .94, 95% CI [.93, .95]).

**Study 3**

**Discriminant Function Analysis**

The canonical discriminant function was statistically significant, χ^2^ (34) = 570.50, *p* < .001, and accounted for 80% of the variance in cultural groups. A total of 94.1% of American and 89% of Chinese participants were classified correctly based on the classification rules. Wilks’ lambda was used to test the statistical significance of each feature of heroes. The value of Wilks’ lambda ranges from 0 to 1, and lower values indicate the greater importance of the variable to the discriminant function. These values identified several excellent predictors for distinguishing between American and Chinese participants.

**Study 6**

**Moderation Effects**

We conducted a series of moderation analyses (PROCESS Model 1; Preacher & Hayes, 2008) to examine the interaction between cultural group and individualism/collectivism. We found there was no significant interaction between cultural group and individualism on identification of civil heroes, *B* = .005, 95%CI [-.05, .06], *t* = .17, *p* = .87.

**Supplementary Table 1**

*Individualism Predicting the Identification of Civil Heroes*

| *Predictors* | *b* | | *SE* | | *t* | | *p* | | 95%CI |
| --- | --- | --- | --- | --- | --- | --- | --- | --- | --- |
| Constant | 17.79 | .14 | | 129.55 | | <.001 | | 17.52, 18.06 | |
| IN  CG  CG x IN | .15  -1.18  .005 | .02  .20  .03 | | 9.04  -5.87  .17 | | <.001  <.001  .87 | | .12, .18  -1.57, -.78  -.05, .06 | |

*Note.* CG = Cultural Group (0 = Chinese; 1= American); IN = Individualism; *R^2^ =* .19*, F* (3, 587) *=* 44.57*, p* < .001. *R^2^ Change =* .00*, F* (1, 587) *=* .03*, p* = .87

We found there was a significant interaction between cultural group and individualism on identification of martial heroes, *B* = .17, 95%CI [.10, .23], *t* = 5.06, *p* <.001. Further tests revealed that the effects of individualism on identification of martial heroes is greater among American participants (*B* = .24, *t* = 9.27, *p* < .001, 95% CI [.19, .29]) than Chinese participants (*B* = .08, *t* = 3.73, *p* < .001, 95% CI [.04, .11].

**Supplementary Table 2.**

*Individualism Predicting the Identification of Martial Heroes*

| *Predictors* | *b* | | *SE* | | *t* | | *p* | | 95%CI |
| --- | --- | --- | --- | --- | --- | --- | --- | --- | --- |
| Constant | 17.15 | .17 | | 101.36 | | <.001 | | 16.81, 17.48 | |
| IN  CG  CG x IN | .08  -1.52  .17 | .02  .25  .03 | | 3.73  -6.15  5.06 | | <.001  <.001  <.001 | | .04, .11  -2.00, -1.03  .10, .23 | |

*Note.* CG = Cultural Group (0 = Chinese; 1= American); IN = Individualism; *R^2^ =* .15*, F* (3, 587) *=* 34.42*, p* < .001; *R^2^ Change =* .04*, F* (1, 587) *=* 25.59*, p* <.001.

We found there was a significant interaction between cultural group and collectivism on identification of civil heroes, *B* = -.09, 95%CI [-.15, -.04], *t* = -3.18, *p* =.002. Further tests revealed that higher levels of collectivism predicted lower levels of identification of civil heroes among American participants (*B* = -.04, *t* = -3.15, *p* = .002, 95% CI [-.07, -.02]) but not among Chinese participants (*B* = .05, *t* = 1.90, *p* = .06, 95% CI [-.002, .10].

**Supplementary Table 3.**

*Collectivism Predicting the Identification of Civil Heroes*

| *Predictors* | *b* | | *SE* | | *t* | | *p* | | 95%CI |
| --- | --- | --- | --- | --- | --- | --- | --- | --- | --- |
| Constant | 17.16 | .15 | | 114.26 | | <.001 | | 16.86, 17.45 | |
| CO  CG  CG x CO | .05  -.12  -.09 | .03  .21  .03 | | 1.90  -.56  -3.18 | | .06  .57  .002 | | -.002, .10  -.52, .29  -.15, -.04 | |

*Note.* CG = Cultural Group (0 = Chinese; 1= American); CO = Collectivism; *R^2^ =* .02*, F* (3, 587) *=* 4.66*, p* = .003. *R^2^ Change =* .02*, F* (1, 587) *=* 10.11*, p* < .001.

We found there was a significant interaction between cultural group and collectivism on identification of martial heroes, *B* = -.08, 95%CI [-.15, -.01], *t* = -2.27, *p* =.02. Further tests revealed that higher levels of collectivism predicted lower levels of identification of martial heroes among American participants (*B* = -.05, *t* = -3.16, *p* = .001, 95% CI [-.09, -.02]) but not among Chinese participants (*B* = .03, *t* = .86, *p* = .39, 95% CI [-.03, .09].

**Supplementary Table 4.**

*Collectivism Predicting the Identification of Martial Heroes*

| *Predictors* | *B* | | *SE* | | *t* | | *p* | | 95%CI |
| --- | --- | --- | --- | --- | --- | --- | --- | --- | --- |
| Constant | 16.82 | .18 | | 92.89 | | <.001 | | 16.47, 17.18 | |
| CO  CG  CG x CO | .03  -.47  -.08 | .03  .25  .04 | | .86  -1.89  -2.27 | | .39  .06  .02 | | -.03, .09  -.96, .02  -.15, -.01 | |

*Note.* CG = Cultural Group (0 = Chinese; 1= American); CO = Collectivism; *R^2^ =* .02*, F* (3, 587) *=* 4.58*, p* = .004. *R^2^ Change =* .009*, F* (1, 587) *=* 5.14*, p* = .02.

**References**

Cheng, C., & Huang, X. (2017). An exploration of courage in Chinese individuals. *The Journal of Positive Psychology, 12*(2), 141-150. https://doi.org/10.1080/17439760.2016.1163406

Jiao, L., Kubler, C. C., & Zhang, W. (2013). *500 common Chinese idioms: An annotated frequency dictionary*. Routledge.

Joo, M., Terzino, K. A., Cross, S. E., Yamaguchi, N., & Ohbuchi, K. I. (2019). How does culture shape conceptions of forgiveness? Evidence from Japan and the United States. *Journal of Cross-Cultural Psychology, 50*(5), 676-702. https://doi.org/10.1177/0022022119845502

Kinsella, E. L., Ritchie, T. D., & Igou, E. R. (2017). On the bravery and courage of heroes: Considering gender. *Heroism Science, 2*(1), 1-14. https://doi.org/10.26736/hs.2017.01.04

Neto, F., & Mullet, E. (2014). A prototype analysis of the Portuguese concept of saudade. *Journal of Cross-Cultural Psychology, 45*(4), 660-670. https://doi.org/10.1177/0022022113518370

Shi, Y., Gregg, A. P., Sedikides, C., & Cai, H. (2021). Lay conceptions of modesty in China: A prototype approach. *Journal of Cross-Cultural Psychology, 52*(2), 155-177. https://doi.org/10.1177/0022022120985318
